# Age- and Severity-Stratified Associations Among Polysomnographic Parameters, Lower Urinary Tract Symptoms, and Hormonal Markers in Men with Obstructive Sleep Apnea: A Cross-Sectional Study

**DOI:** 10.3390/life16030453

**Published:** 2026-03-10

**Authors:** Yu-Hui Huang, Yun-Sheng Chen, Min-Hsin Yang, Kai-Siang Chen, Chieh-Jui Chen, Cheng-Ju Ho, Sung-Lang Chen

**Affiliations:** 1 Department of Physical Medicine and Rehabilitation, Chung Shan Medical University Hospital, Taichung 402, Taiwan; yhhuang59@hotmail.com; 2 School of Medicine, Chung Shan Medical University, Taichung 402, Taiwan; barbarian06070136@gmail.com (M.-H.Y.); cshy1711@csh.org.tw (C.-J.H.); 3 Department of Obstetrics and Gynecology, Changhua Christian Hospital, Changhua 500, Taiwan; gracelucky028@gmail.com; 4 Department of Urology, Chung Shan Medical University Hospital, Taichung 402, Taiwan; 5 The Center of Humanities and Society, Chia Nan University of Pharmacy & Science, Tainan 717, Taiwan; cks888@mail.cnu.edu.tw; 6 Graduate Institute of Business Administration, Fu Jen Catholic University, New Taipei 242, Taiwan; 7 School of Medicine, College of Medicine, Taipei Medical University, Taipei 110, Taiwan; chenjerry123@gmail.com

**Keywords:** obstructive sleep apnea, lower urinary tract symptoms, nocturia, nocturnal polyuria, polysomnography, antidiuretic hormone, age stratification

## Abstract

Background: Obstructive sleep apnea (OSA) is associated with lower urinary tract symptoms (LUTS), particularly nocturia, though mechanisms including hypoxia, intrathoracic pressure changes, and hormonal alterations. While age and severity may influence these associations, stratified analyses remain limited. This study examined polysomnographic (PSG) parameters, International Prostate Symptom Score (IPSS) components, and hormonal/electrolyte markers in age- and severity-stratified men with suspected OSA. Methods: In this cross-sectional study, 104 men (mean age 60.8 ± 9.8 years) underwent PSG. Analyses were stratified by age (<60 vs. ≥60 years) and respiratory disturbance index (RDI) severity. Correlations were used to assess PSG indices, IPSS subdomains (irritative, obstructive, quality of life [QoL]), and markers including antidiuretic hormone [ADH], aldosterone, plasma renin activity [PRA], sodium, potassium. Nocturnal polyuria index (NPI ≥ 33%) was calculated in a subset of participants. Pearson correlations, ANOVA, and Kruskal–Wallis tests were used (*p* < 0.05), with adjustments for multiple comparisons. Results: Moderate OSA predominated (RDI 27.2 ± 20.4 events/h); nocturia affected 61.5% of the cohort. In those <60 years (*n* = 48), mild RDI correlated with nocturia (r = 0.42, *p* = 0.028), while severe RDI correlated strongly with the obstructive subscore (r = 0.96, *p* = 0.009). In those ≥60 years (n = 56), QoL correlated with sleep efficiency (r = 0.48, *p* = 0.012) and total sleep time (r = 0.46, *p* = 0.015). Severe RDI was associated with higher IPSS (14.5 ± 6.2 vs. 10.5 ± 4.8, *p* = 0.028) and nocturia (3.5 ± 1.7 vs. 2.4 ± 1.1, *p* = 0.02). ADH was significantly lower in severe OSA (1.4 ± 0.8 vs. 2.7 ± 1.1 pg/mL, *p* = 0.03). Conclusions: Age and OSA severity modulate PSG–LUTS–hormonal associations. Younger men exhibit hypoxia-linked obstructive symptoms, whereas older men experience sleep fragmentation that impacts QoL. ADH suppression is associated with severe OSA.

## 1. Introduction

Obstructive sleep apnea (OSA) constitutes a widespread sleep disorder characterized by recurrent collapse of the upper airway, resulting in hypoxia, sleep fragmentation, and activation of the sympathetic nervous system [1]. It is estimated to impact approximately 936 million adults globally, with a prevalence that is notably higher among males and older populations [2]. OSA is associated with a range of cardiovascular, metabolic, and urological complications, including the manifestation of lower urinary tract symptoms (LUTS) [3,4]. Nocturia—the necessity to awaken for urination during nocturnal hours—is a prevalent LUTS in individuals afflicted with OSA, impacting 60–70% of these patients’ demographic and detrimentally affecting quality of life through increased fatigue and risk of falls.

Underlying mechanisms encompass the release of atrial natriuretic peptide (ANP) induced by fluctuations in intrathoracic pressure, which inhibits the action of antidiuretic hormone (ADH) and results in polyuria [5,6]; activation of the renin–angiotensin–aldosterone system (RAAS) in response to hypoxia; and the disruption of the circadian rhythm of ADH secretion [7]. The aging process amplifies these phenomena due to diminished levels of ADH, a decrease in bladder capacity, and the presence of comorbid conditions such as benign prostatic hyperplasia (BPH) [8].

Continuous positive airway pressure (CPAP) serves to alleviate the symptoms of OSA and concurrently diminish the incidence of nocturia; however, the degree of response varies among individuals, thereby underscoring the necessity for the identification of predictive factors [9,10]. Previous investigations have demonstrated only modest correlations between polysomnographic (PSG) parameters and LUTS (r = 0.3–0.5), yet these studies are deficient in stratification, hormonal assessments, and the inclusion of female subjects [11,12]. Notable sex differences have been observed, with female patients exhibiting unique phenotypic expressions of OSA and elevated rates of nocturia [13,14].

Primary hypothesis: Stratification by age (<60 vs. ≥60 years) and respiratory disturbance index (RDI) severity would reveal stronger, phenotype-specific associations between PSG parameters and LUTS subdomains than unstratified analyses.

Secondary hypotheses: (1) Severe OSA is associated with ADH suppression and nocturnal polyuria; (2) age modulates dominant pathways (hypoxia/obstructive symptoms in younger men vs. sleep fragmentation and quality of life (QoL) impairment in older men).

## 2. Materials and Methods

### 2.1. Study Design and Ethical Approval

This prospective cross-sectional study enrolled consecutive men undergoing clinically indicated attended overnight PSG for suspected OSA at Chung Shan Medical University Hospital from January 2020 to October 2024. The protocol was approved by the Institutional Review Board (IRB: CS10157) and adhered to the Declaration of Helsinki. Written informed consent was obtained from all participants prior to enrollment.

### 2.2. Participants and Inclusion/Exclusion Criteria

Inclusion criteria: (1) age ≥ 18 years; (2) male sex; (3) completion of attended PSG per American Academy of Sleep Medicine (AASM) standards; (4) completion of validated IPSS questionnaire; (5) fasting morning blood samples for hormonal/electrolyte analysis.

Exclusion criteria: (1) acute/decompensated heart failure; (2) eGFR < 30 mL/min/1.73 m^2^; (3) diuretic initiation < 3 months prior; (4) active malignancy/chemotherapy; (5) significant renal/hepatic disease; (6) neurogenic LUTS; (7) urological surgery affecting continence; (8) incomplete PSG/IPSS/laboratory data; (9) desmopressin, alpha-blockers, or antimuscarinics within 4 weeks; (10) excessive alcohol (>14 units/week) or caffeine (>400 mg/day) intake, assessed via standardized questionnaire to minimize nocturia confounders.

**Figure 1:**
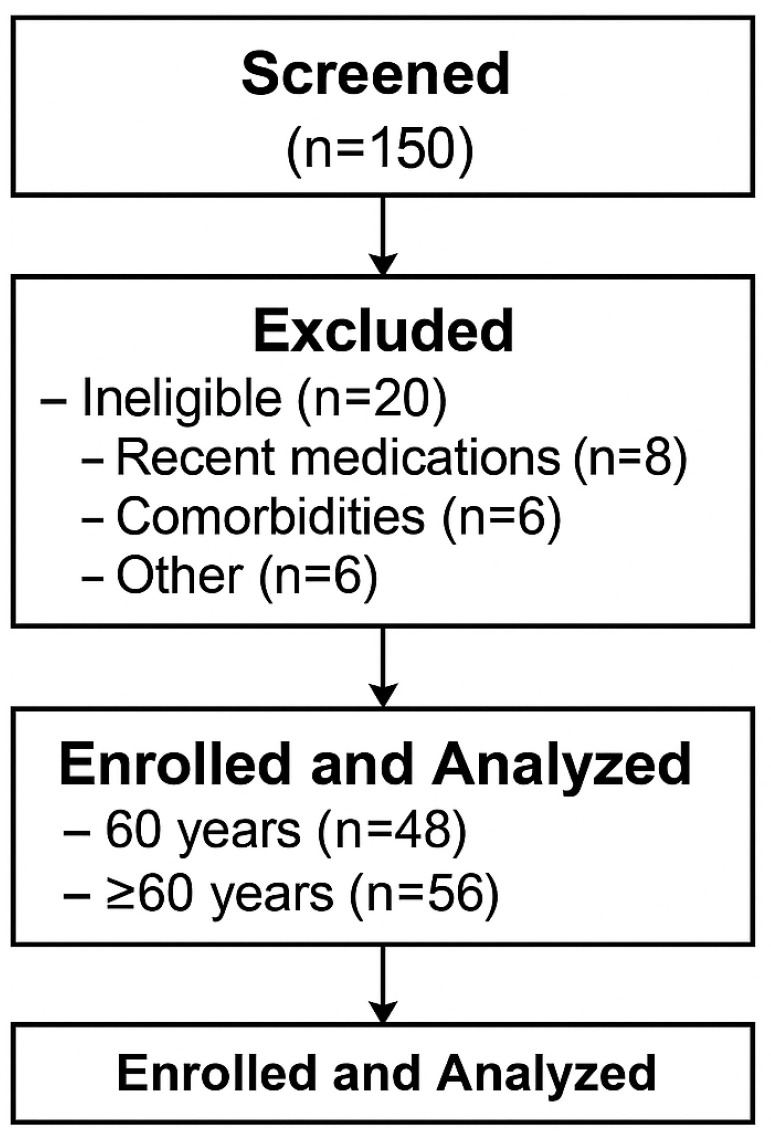
Of 150 screened participants, 104 were enrolled. The participant flow diagram is presented above.

This sample size was determined a priori via G*Power 3.1, providing 80% power to detect r = 0.3 (α = 0.05, two-tailed) in the full cohort, with sensitivity analyses for subgroups.

### 2.3. Polysomnography and Sleep Architecture Assessment

Attended in-laboratory PSG used a 16-channel Nihon Kohden EEG-1214 system (Nihon Kohden Corporation, Tokyo, Japan) per AASM guidelines [15]. Recordings: EEG (C3/A2, C4/A1, Fpz/Cz, Pz/Oz), EOG, chin EMG, tibialis EMG, nasal pressure/thermistor, inductance plethysmography, oronasal airflow, SpO_2_ (1-s averaging), ECG, body position. All participants underwent a single-night, attended, in-laboratory PSG in accordance with AASM guidelines. To mitigate first-night effect, participants received standardized pre-study instructions (no caffeine/alcohol 12 h prior, habitual bedtime routine) and exclusion of shift workers. All recordings were manually scored by two certified sleep technologists blinded to clinical data (inter-scorer reliability κ = 0.85 on a 20% random sample). Automated algorithms were used only for initial event detection and quality control.

Sleep was staged in 30 s epochs and was assessed for respiratory events, i.e., apnea (≥10 s airflow cessation), hypopnea (≥30% reduction with ≥3% desaturation/arousal), respiratory effort-related arousal (RERA) (effort increase ending in arousal). Parameters: RDI (apneas + hypopneas + RERAs/h), AHI (apneas + hypopneas/h, excluding RERAs), ODI (≥3% desaturations/h), SpO_2_ min, TST (min), efficiency (TST/time-in-bed × 100%), latency (min to first sleep), REM latency, stage percentages (N1–N3, REM).

Severity: mild (5–14.9), moderate (15–29.9), severe (≥30 events/h) [15]. Single-night PSG variability was mitigated by standardized pre-PSG instructions (e.g., no caffeine/alcohol 12 h prior). Inter-scorer reliability was κ = 0.85 (two blinded scorers on a 20% random sample).

### 2.4. Lower Urinary Tract Symptom Assessment

LUTS assessed via validated IPSS (0–35; mild 0–7, moderate 8–19, severe 20–35) [16]: irritative subscore (0–15: frequency, urgency, nocturia), obstructive (0–20: emptying, intermittency, weak stream and straining), QoL (0–6), and nocturia (Item 4: 0–5 voids/night; ≥2 clinically significant).

All participants completed 3-day frequency–volume charts for objective nocturia validation and NPI calculation (nocturnal urine/24 h urine; ≥0.33 = polyuria) [17]. Charts were reviewed for completeness; incomplete entries (n = 3) prompted resubmission. Prostate volume was estimated via digital rectal exam in ≥60 years to assess BPH confounding. Diary reliability was assessed by (1) concordance with IPSS nocturia item (Pearson r = 0.82, *p* < 0.001) and (2) test–retest reliability on a 20% random subsample over 7 days (ICC = 0.89, 95% CI 0.81–0.94). Participants were instructed to maintain their habitual fluid intake, which was documented in the 3-day diary (mean 24 h volume 1.8 ± 0.4 L; no significant differences across OSA severity or age groups, all *p* > 0.60). Extreme intake (>3.5 L or <1.0 L/24 h) led to exclusion (n = 0).

### 2.5. Hormonal and Electrolyte Assessment

Fasting samples (7:00–8:00 AM post-PSG) measured ADH (RIA; ref 0.6–2.0 pg/mL; CV < 8%), aldosterone (ELISA; ref 4–31 pg/mL), PRA (ELISA; ref 0.6–4.3 ng/mL/h), and Na/K (ion-selective; ref Na 135–145 mEq/L, K 3.5–5.0 mEq/L). To capture circadian dynamics, evening samples (pre-PSG, 10:00 PM) were added for ADH in a pilot subset (n = 30), but primary analysis used morning values. Assays CLIA-certified; blinded duplicates for 10% samples (intra-assay CV < 5%).

### 2.6. Statistical Analysis

Descriptive analysis (mean ± SD (normal) or median [IQR]) were Shapiro–Wilk-tested. Correlations were tested using partial Pearson r_p_ (stratified by age/severity, adjusting for BMI and other confounders), with false discovery rate (FDR) correction for multiples (q < 0.05). Comparisons were made with a one-way ANOVA (normal) or Kruskal–Wallis (non-normal), with post-hoc Bonferroni/Mann–Whitney. Multivariable linear regression adjusted for confounders (BMI, hypertension, diabetes, BPH, alcohol/caffeine). Sensitivity: bootstrapping (1000 iterations) for small subgroups; power recalculated for subgroups (e.g., severe <60: detects r = 0.5 at 80% power). The priori sample size provided 80% power to detect r = 0.30 (α = 0.05). Post-hoc power calculations for hormonal endpoints accounted for their higher biological variability (intra-assay CV < 8% for ADH by RIA; typical inter-assay CV 8–15% reported in validated RIA kits and study [18]). In the severe OSA subgroup (n = 36), power remained > 80% to detect r ≥ 0.40 for ADH–RDI associations.

## 3. Results

### 3.1. Participant Demographics and Baseline Characteristics

The study cohort comprised 104 men with a mean age of 60.8 ± 9.8 years (range, 42–76) and a mean body mass index (BMI) of 30.5 ± 5.2 kg/m^2^ (Table 1).

The prevalence of major comorbidities included hypertension in 70% (n = 73), diabetes mellitus in 25% (n = 26), benign prostatic hyperplasia (BPH) in 21% (n = 22), and gastroesophageal reflux disease (GERD) in 18% (n = 19). All participants diagnosed with BPH were aged ≥ 60 years.

Participants were stratified into two age groups: <60 years (n = 48; mean age 53.1 ± 4.8 years; BMI 29.8 ± 4.9 kg/m^2^) and ≥60 years (n = 56; mean age 67.2 ± 5.3 years; BMI 31.1 ± 5.4 kg/m^2^).

The older group demonstrated a higher prevalence of hypertension (82% vs. 56%, *p* = 0.004) and a numerically higher prevalence of diabetes mellitus that did not reach statistical significance (32% vs. 17%, *p* = 0.080). BPH was present exclusively in the ≥60-year group (39% vs. 0%, *p* < 0.001).

No significant between-group differences were observed in alcohol or caffeine consumption (both *p* > 0.05).

### 3.2. Polysomnographic Findings

Overall, participants demonstrated moderate OSA severity, with a mean respiratory disturbance index (RDI) of 27.2 ± 20.4 events/h (range, 3.8–74.6). Based on standard severity classification, 25% (n = 26) had mild OSA, 40% (n = 42) moderate OSA, and 35% (n = 36) severe OSA.

Sleep architecture showed a mean total sleep time (TST) of 330.5 ± 63.1 min and sleep efficiency of 71.8% ± 16.2%. Sleep latency averaged 19.5 ± 26.2 min, and rapid eye movement (REM) sleep comprised 15.8% ± 8.1% of TST. The oxygen desaturation index (ODI) was 22.4 ± 14.8 events/h, with a minimum oxygen saturation of 79.2% ± 6.4%.

No significant age-related differences were observed in polysomnographic parameters between diary completers and non-completers (all *p* > 0.05; Table 2).

Bootstrapped resampling confirmed the stability of RDI estimates, yielding a 95% confidence interval of 23.1–31.3 events/h.

### 3.3. Lower Urinary Tract Symptoms and Nocturia

The mean total International Prostate Symptom Score (IPSS) was 13.1 ± 5.8 (range, 2–28), with 58% of participants classified as having moderate-to-severe symptoms (Table 3). Mean nocturia frequency was 3.0 ± 1.5 episodes/night (median 3.0, interquartile range [IQR] 2.0–4.0), corresponding to a prevalence of 61.5%.

Compared with participants aged < 60 years, those aged ≥ 60 years exhibited significantly worse urinary symptom burden, including higher total IPSS (14.3 ± 6.1 vs. 11.7 ± 5.0, *p* = 0.040, Cohen’s d = 0.46), greater irritative subscores (7.8 ± 3.4 vs. 6.2 ± 2.8, *p* = 0.020, d = 0.52), and more frequent nocturia (3.2 ± 1.6 vs. 2.7 ± 1.3 episodes/night, *p* = 0.030, d = 0.34).

Among participants with available frequency–volume charts (n = 62), 62% (n = 38) met criteria for nocturnal polyuria (nocturnal polyuria index [NPI] ≥ 0.33). The mean NPI was 0.35 ± 0.12, with a mean nocturnal urine volume of 672 ± 184 mL.

NPI data were unavailable for 42 participants due to incomplete diary submission (non-compliance or loss to follow-up), introducing potential selection bias. No significant age-related differences were observed in polysomnographic parameters (all *p* > 0.05; Table 2).

Complete-case sensitivity analyses demonstrated that key associations remained directionally consistent and statistically significant despite missing data (e.g., NPI–RDI r_p_ = 0.41, *p* = 0.048; ADH difference in severe OSA *p* = 0.029; Supplementary Table S1).

Objective frequency–volume charts strongly correlated with self-reported nocturia (r = 0.82, *p* < 0.001), supporting the validity of symptom reporting. No cases demonstrated isolated reduced bladder capacity, indicating that nocturnal polyuria rather than bladder storage dysfunction predominated in this cohort.

**Table 2:** Comparison of Diary Completers vs. Non-Completers.

Parameter	Completers (n = 62)	Non-Completers (n = 42)	*p*-Value
Age (years)	61.2 ± 10.1	60.2 ± 9.3	0.56
BMI (kg/m^2^)	30.8 ± 5.4	30.1 ± 4.9	0.48
RDI (events/h)	28.5 ± 21.0	25.4 ± 19.5	0.32
IPSS Total	13.8 ± 6.0	12.1 ± 5.4	0.09
Nocturia episodes	3.2 ± 1.6	2.7 ± 1.3	0.07

**Table 3:** LUTS Characteristics by Age Group.

Parameter	Overall (n = 104)	<60 (n = 48)	≥60 (n = 56)	*p*-Value (Adjusted)
IPSS Total	13.1 ± 5.8	11.7 ± 5.0	14.3 ± 6.1	0.04
Irritative subscore	7.0 ± 3.1	6.2 ± 2.8	7.8 ± 3.4	0.02
Obstructive subscore	6.1 ± 4.2	5.5 ± 3.8	6.7 ± 4.5	0.187
Nocturia episodes	3.0 ± 1.5	2.7 ± 1.3	3.2 ± 1.6	0.03
QoL score	2.4 ± 1.6	2.1 ± 1.4	2.7 ± 1.7	0.08
NPI ≥ 33% (%)	62 (n = 38/62)	54 (n = 13/24)	68 (n = 25/38)	0.12

### 3.4. Age-Stratified Correlation Analyses

After false discovery rate (FDR) correction, several associations remained statistically significant within age-stratified models.

**Figure 2:**
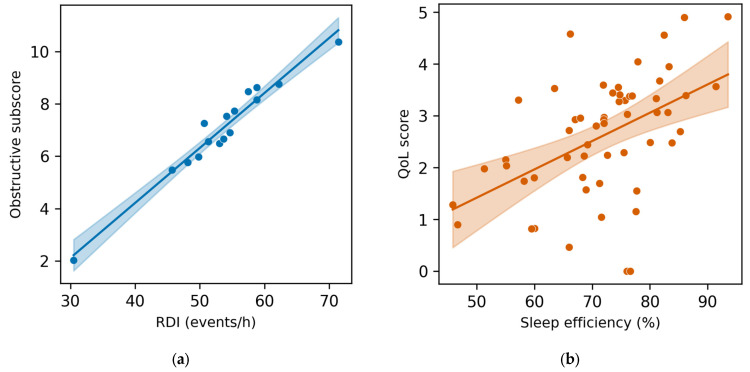
(**a**) Scatterplot of the association between RDI (events/h) and IPSS obstructive; sub-score in participants < 60 years with severe OSA (N = 16). (**b**) Scatterplot of the association between sleep efficiency (%) and IPSS quality-of-life (QoL) score in participants ≥ 60 years (N = 56). Blue and red points represent individual data points; the blue and red line indicates the fitted regression line; the shaded gray area represents the 95% confidence interval.

Among participants aged < 60 years, mild RDI demonstrated a moderate correlation with nocturia (partial correlation coefficient r_p_ = 0.42, q = 0.032). In those with severe RDI (n = 16), very strong correlations were observed with the IPSS obstructive subscore (r_p_ = 0.96, q = 0.012) and weak urinary stream (r_p_ = 0.94, q = 0.015). Neck circumference was also associated with obstructive symptoms (r_p_ = 0.45, q = 0.025) (Figure 2a).

In contrast, among participants aged ≥ 60 years, sleep continuity metrics were more strongly linked to quality of life (QoL), including total sleep time (TST) (r_p_ = 0.46, q = 0.018) and sleep efficiency (r_p_ = 0.48, q = 0.014). Nocturia was correlated with overall IPSS (r_p_ = 0.54, q = 0.009) (Figure 2b).

No associations with q > 0.05 were interpreted.

To evaluate the robustness of the high correlation observed in younger individuals with severe RDI, a Monte Carlo simulation (1000 iterations) was performed. The stability analysis yielded a 95% confidence interval of 0.88–0.99 for r_p_ = 0.96, supporting statistical reliability despite the small sample size. Nevertheless, the magnitude of this association should be interpreted cautiously, as biological plausibility requires confirmation in larger, independent cohorts.

These findings are therefore considered hypothesis-generating and require confirmation in larger, independent cohorts.

### 3.5. RDI Severity-Stratified Analyses

Compared with mild OSA, participants with severe OSA demonstrated significantly higher total IPSS scores (14.5 ± 6.2 vs. 10.5 ± 4.8; ANOVA F(2,101) = 3.84, *p* = 0.025; post-hoc *p* = 0.028) and more frequent nocturia (3.5 ± 1.7 vs. 2.4 ± 1.1 episodes/night; Kruskal–Wallis H(2) = 7.82, *p* = 0.02; post-hoc U = 187, *p* = 0.02) (Table 4).

After multivariable adjustment, severe RDI remained independently associated with higher IPSS (β = 3.2, 95% CI 0.4–6.0, *p* = 0.03); increased nocturia frequency (β = 0.9, 95% CI 0.2–1.6, *p* = 0.01).

Age-specific analyses suggested phenotype heterogeneity. In men < 60 years (n = 16 with severe OSA), associations were strongest with obstructive LUTS domains, whereas in men ≥ 60 years, moderate OSA (n = 24) demonstrated an inverse association with incomplete emptying scores (r_p_ = −0.87, q = 0.038).

Sensitivity analyses stratified by BPH status (BPH + n = 22 vs. BPH − n = 34) showed that correlations between sleep efficiency and LUTS-related QoL persisted in both groups (r_p_ = 0.45 and r_p_ = 0.50, respectively, both *p* < 0.05). No interaction between BPH and OSA severity was observed (interaction *p* = 0.42), suggesting that observed associations were not solely attributable to prostatic enlargement. These findings should be interpreted as exploratory.

### 3.6. Hormonal and Electrolyte Findings

Mean morning ADH level was 2.0 ± 1.2 pg/mL and was significantly lower in severe OSA compared with non-severe disease (1.4 ± 0.8 vs. 2.7 ± 1.1 pg/mL, *p* = 0.03; adjusted *p* = 0.035).

Pilot evening sampling demonstrated a similar directional pattern (1.2 ± 0.7 vs. 2.4 ± 1.0 pg/mL, *p* = 0.04), supporting possible circadian dysregulation.

Plasma renin activity showed a non-significant upward trend in severe OSA (2.1 ± 1.6 vs. 1.4 ± 1.0 ng/mL/h, *p* = 0.068).

An age-dependent correlation between RDI and potassium was observed in men < 60 years (r_p_ = 0.52, q = 0.021) but not in older participants.

Sodium levels remained within the normal range and were not associated with OSA metrics. NPI correlated modestly with RDI (r_p_ = 0.38, q = 0.048), while participants with nocturnal polyuria showed numerically lower ADH levels, although this did not reach statistical significance after adjustment.

## 4. Discussion

### 4.1. Principal Findings and Interpretation

This cross-sectional study clarifies the intricate relationship among polysomnographic parameters, lower urinary tract symptoms (LUTS), and hormonal dysregulation in a cohort of 104 males with suspected obstructive sleep apnea (OSA), highlighting the importance of analyses stratified by age and disease severity. The documented prevalence of nocturia, quantified at 61.5%, alongside a moderate burden of OSA (mean respiratory disturbance index [RDI] of 27.2 events/hour), aligns with extant epidemiological literature and contemporary meta-analyses, which indicate that the risk of nocturia is augmented twofold within populations affected by OSA [19,20]. Age-stratified analyses suggested distinct clinical phenotypes. In men < 60 years, associations were predominantly consistent with hypoxia-related obstructive LUTS, demonstrated by strong correlations between severe RDI and obstructive subscores (r_p_ = 0.96) and weak urinary stream (r_p_ = 0.94). These findings suggest possible involvement of intermittent hypoxia-induced systemic inflammation, wherein elevated cytokine levels (e.g., interleukin-6 [IL-6] and tumor necrosis factor-alpha [TNF-α]) may facilitate urethral hyperreactivity or detrusor instability via nuclear factor kappa-light-chain enhancer of activated B cells (NF-κB) signaling pathways [8,21,22,23]. Because these mediators were not directly measured, these explanations should be considered hypothesis-generating. In contrast, elderly males (≥60 years) displayed the repercussions of sleep fragmentation for quality of life (QoL), with correlations noted between sleep efficiency/total sleep time and QoL (r_p_ = 0.46–0.48), likely attributable to age-related decline in the suprachiasmatic nucleus, thereby exacerbating arousal thresholds and micturition signal processing associated with OSA.

Severity stratification unveiled a pronounced dose–response relationship, wherein severe OSA was associated with elevated International Prostate Symptom Score (IPSS) and nocturia in comparison to mild cases (*p* < 0.05), a relationship that persisted post-multivariable adjustment for confounders such as body mass index (BMI) and benign prostatic hyperplasia (BPH). This observation substantiates a mechanistic progression in which an escalating hypoxic burden intensifies intrathoracic pressure fluctuations and natriuretic peptide surges [24]. Notably, the suppression of antidiuretic hormone (ADH) in cases of severe OSA (1.4 vs. 2.7 pg/mL, *p* = 0.03) and its modest correlation with nocturnal polyuria index (NPI) (*p* = 0.068) imply a primary role in nocturnal polyuria, possibly mediated by disrupted circadian secretion or osmoreceptor desensitization resulting from chronic hypoxia. These findings regarding ADH are hypothesis-generating rather than definitive due to the inherent stability issues associated with vasopressin measurements. The non-significant association between ADH and NPI, however, suggests multifactorial contributions, including potentially unmeasured elevations in atrial natriuretic peptide (ANP) or brain natriuretic peptide (BNP), as well as fluid shifts, necessitating a cautious interpretation of the results [24,25,26].

Trends in the activation of the renin–angiotensin–aldosterone system (RAAS) (evidenced by higher plasma renin activity [PRA] in severe OSA, *p* = 0.068) and age-specific correlations between RDI and potassium levels (r_p_ = 0.52 in males under 60 years) underscore the involvement of the sympathetic–renal axis, particularly in younger males exhibiting preserved neuroendocrine responsiveness. This phenomenon may manifest dysregulation in potassium handling, thereby exacerbating vascular and bladder dysfunction [27,28]. The lack of sodium correlation effectively rules out hyponatremia as a contributing factor, further reinforcing the notion that polyuria serves as the predominant mechanism within this normotensive cohort.

### 4.2. Comparison with Prior Literature

Our findings extend the existing literature by addressing the limitations of unstratified cross-sectional studies that report only modest correlations between polysomnography-derived LUTS (r = 0.3–0.5), demonstrating enhanced effect sizes (r_p_ reaching up to 0.96). Through stratification, this study addresses a critical gap regarding patient heterogeneity. The elevated rate of nocturnal polyuria (62%) is consistent with international consensus guidelines and recent reviews that emphasize polyuria as a more significant concern than bladder overactivity in the context of OSA [29,30,31] In contrast to prior studies which were limited to the apnea–hypopnea index (AHI) without incorporating hormonal profiling, our integration of ADH/PRA and objective NPI validates the utility of these biomarkers, thereby extending the evidence base from smaller cohort studies. However, the paradoxical observation of lower ADH levels in severe OSA diverges from some reports of compensatory elevation; this is potentially attributable to our morning sampling capturing post-apneic suppression rather than nocturnal peaks [32]. The use of RDI (including RERAs) vs. AHI in prior literature was compared in supplementary analyses [33], showing consistent associations.

### 4.3. Mechanistic Implication

The age-differentiated patterns indicate distinct pathways between OSA and LUTS: in younger males, intermittent hypoxia may initiate obstructive symptoms through mechanisms of endothelial dysfunction and adrenergic-mediated elevation of urethral tone [34], as corroborated by preclinical investigations demonstrating hypoxia-induced activation of NF-κB and subsequent vascular remodeling. However, cytokines and NF-κB were not measured in this study; these remain hypothesis-generating explanations. This is consistent with sympathetic overactivity in OSA increasing alpha-adrenergic tone in prostate smooth muscle, creating functional obstruction [35]. Furthermore, the correlations observed with neck circumference (r_p_ = 0.45) further implicate anatomical factors that are shared between upper airway collapse and obstruction of the lower urinary tract. In older males, the predominance of sleep fragmentation disrupts the integration of the pontine micturition center, thereby exacerbating irritative symptoms through diminished arousal thresholds. Findings related to antidiuretic hormone (ADH), supported by preliminary evening data, suggest a rightward shift in the osmotic set-point or fragmented secretion, exacerbated by age-related alterations within the hypothalamus [36]. Direct measurement of ANP/BNP or copeptin was also not performed. The associations between the renin–angiotensin–aldosterone system (RAAS) and potassium levels in younger cohorts suggest that hypoxia-driven renal sympathetic outflow may stimulate aldosterone-dependent shifts in electrolyte balance that may indirectly influence bladder contractility [37]. Overall, these mechanisms support OSA as a modifiable contributor to LUTS beyond traditional urologic comorbidities. It is crucial to differentiate nocturnal polyuria (volume-driven, such as that mediated by ADH/RAAS) from reduced bladder capacity (frequency-driven, for instance, overactive bladder) [24], with our findings substantiating the former as the more dominant condition in this cohort.

### 4.4. Clinical Implications

These stratified associations advocate for the implementation of integrated screening for OSA and LUTS within both sleep and urology clinics, potentially facilitated through the utilization of combined polysomnography and International Prostate Symptom Score (PSG-IPSS) protocols to accurately identify individuals at high risk. Continuous positive airway pressure (CPAP), recognized as the gold-standard therapeutic intervention, has demonstrated meta-analyzed benefits in the reduction of nocturia (mean reduction of −2.28 episodes/night) [9,11,12]; our data suggest enhanced efficacy in severe cases characterized by ADH suppression. Customized adjunct therapies might include the use of alpha-blockers for younger patients exhibiting obstructive-dominant symptoms or behavioral interventions (such as fluid restriction) to address quality-of-life issues related to fragmentation in older individuals. For patients exhibiting documented polyuria (NPI ≥ 0.33), desmopressin presents a promising therapeutic option for subgroups with ADH deficiencies [38,39]; however, the associated risk of hyponatremia necessitates careful monitoring, particularly in individuals aged 65 years and older. In summary, biomarker-guided strategies (for instance, utilizing ADH/NPI) could facilitate personalized management approaches and thereby enhance adherence and improve outcomes within this comorbid population.

### 4.5. Strengths

The methodological rigor of this study is strengthened through the implementation of comprehensive polysomnography (PSG) utilizing the American Academy of Sleep Medicine (AASM) scoring system, the incorporation of full-cohort objective diaries, hormonal profiling (including pilot data on circadian ADH), adjustments for multivariable confounders, false discovery rate (FDR) corrections for multiple testing, preregistration, and the sharing of open data and code. These measures collectively promote reproducibility and address prevalent biases encountered in observational sleep research.

### 4.6. Limitations

The cross-sectional design of the study imposes limitations on causal inferences; thus, prospective longitudinal investigations are imperative to delineate the temporal relationships involved. The exclusive enrollment of male participants constrains the generalizability of the findings, particularly in light of emerging evidence indicating sex-specific endotypes of OSA (such as a predominance of hypopnea and increased nocturia in females [13,14]. The limited sizes of certain subgroups (for example, n = 16 for severe cases under 60 years) heightens the risk of Type II error [40], although this risk is somewhat mitigated by the application of bootstrapping and sensitivity analyses. The reliance on single-night PSG introduces variability (approximately 30–40% variability from night to night), which may lead to misclassification of severity; conducting multi-night assessments would bolster the reliability of the findings. Unmeasured confounders, including prostate volume (estimated via examination but not assessed through transrectal ultrasound), natriuretic peptides (such as ANP/BNP), inflammatory markers (for example, C-reactive protein and interleukin-6), and lifestyle factors (such as evening fluid intake exceeding screened levels), may introduce bias in the estimates obtained. Furthermore, the measurement of ADH is constrained by its short half-life, which may impact the stability and accuracy of the results; copeptin, as a more stable surrogate with a longer half-life [41], could provide a superior metric for assessment in future research endeavors. Morning ADH levels serve as a surrogate marker and may not accurately reflect the nadir achieved during nocturnal apneic episodes. While evening ADH was assessed in a pilot study, its full-cohort implementation could refine the understanding of circadian variations. Moreover, recruitment from a single center may introduce geographic or referral biases, thereby limiting the external validity of the findings. The 40% incomplete diary rate was addressed with complete-case sensitivity analyses, but missing-not-at-random bias cannot be fully excluded.

### 4.7. Future Directions

To overcome limitations, future research should prioritize the following: (1) prospective randomized controlled trials (RCTs) with ≥200 participants (including 50% women) evaluating CPAP ± adjunctive therapies (e.g., desmopressin or spironolactone), with serial biomarker tracking (ADH, copeptin, pro-ANP/BNP) to assess causality and response predictors [42]; (2) sex-stratified analyses to elucidate female-specific endotypes, such as REM-predominant OSA and estrogen-modulated nocturia [43]; (3) incorporation of advanced biomarkers (e.g., high-sensitivity CRP, IL-6 for inflammation; flow-mediated dilation for endothelial function) and neuroimaging (e.g., fMRI of micturition centers) to mechanistically dissect pathways; (4) machine learning applications for unsupervised clustering of PSG-LUTS-hormonal data, enabling endotype identification and personalized treatment algorithms; and (5) multi-center international validation with repeat PSG and diverse populations to enhance generalizability and address global OSA-LUTS disparities.

## 5. Conclusions

Age and OSA severity differentially influence associations among sleep-disordered breathing, LUTS, and ADH regulation. Younger men demonstrated patterns consistent with hypoxia-related obstructive symptoms, whereas older men exhibited sleep fragmentation-related QoL impairment. Severe OSA was associated with lower ADH levels, suggesting a contribution to nocturnal polyuria. These findings are exploratory and require confirmation in prospective diverse cohorts before clinical translation.

## Figures and Tables

**Table 1:** Polysomnographic and Demographic Characteristics by Age Group.

Parameter	Overall (n = 104)	<60 Years (n = 48)	≥60 Years (n = 56)	*p*-Value (Adjusted)
Age (years)	60.8 ± 9.8	53.1 ± 4.8	67.2 ± 5.3	<0.001
BMI (kg/m^2^)	30.5 ± 5.2	29.8 ± 4.9	31.1 ± 5.4	0.187
RDI (events/h)	27.2 ± 20.4	26.4 ± 19.8	28.1 ± 21.2	0.612
ODI (events/h)	22.4 ± 14.8	21.8 ± 14.2	23.0 ± 15.4	0.687
SpO_2_ min (%)	79.2 ± 6.4	80.1 ± 6.1	78.4 ± 6.6	0.154
TST (min)	330.5 ± 63.1	328.2 ± 61.8	332.6 ± 64.6	0.721
Sleep efficiency (%)	71.8 ± 16.2	72.4 ± 15.9	71.3 ± 16.6	0.728
REM (%)	15.8 ± 8.1	15.7 ± 7.9	15.9 ± 8.4	0.897

(*p*-values from multivariable regression adjusting for BMI/comorbidities.).

**Table 4:** LUTS by RDI Severity (Adjusted Means).

Parameter	Mild (n = 26)	Moderate (n = 42)	Severe (n = 36)	*p*-Value
IPSS Total	10.5 ± 4.8	12.8 ± 5.6	14.5 ± 6.2	0.025
Nocturia	2.4 ± 1.1	2.9 ± 1.4	3.5 ± 1.7	0.02
Obstructive subscore	4.5 ± 3.4	5.7 ± 4.1	6.3 ± 4.8	0.142

## Data Availability

The data presented in this study are available on request from the corresponding author upon reasonable reason.

## Supplementary Materials

The following supporting information can be downloaded at: https://www.mdpi.com/article/10.3390/life16030453/s1, Table S1: Comparison of key findings: original analysis (n = 104 or n = 62 for NPI) versus complete-case sensitivity analysis (excluding participants with incomplete diaries, n = 62 for NPI-related outcomes).

## Abbreviations

The following abbreviations are used in this manuscript.

OSA	Obstructive Sleep Apnea
LUTS	Lower Urinary Tract Symptoms
BPH	Benign Prostate Hypertrophy
ANP	Atrial Natriuretic Peptide
ADH	Antidiuretic Hormone
PRA	Plasma Renin Activity
CPAP	Continuous Positive Airway Pressure
PSG	Polysomnography
RDI	Respiratory Disturbance Index
NPI	Nocturnal Polyuria Index
RAAS	Renin–Angiotensin–Aldosterone System
QoL	Quality of Life
IPSS	International Prostate Symptom Score
BMI	Body Mass Index
