# Supplementary material for: Age- and Severity-Stratified Associations Among Polysomnographic Parameters, Lower Urinary Tract Symptoms, and Hormonal Markers in Men with Obstructive Sleep Apnea: A Cross-Sectional Study
Source: Life (Basel). 2026 Mar 10;16(3):453. doi: 10.3390/life16030453 (PMC13027505; doi:10.3390/life16030453)
Supplement: Supplementary file 1 [file life-16-00453-s001.zip › life-4138546-supplementary.pdf]

Supplementary Materials

Supplementary Table S1. Comparison of key findings: original analysis (n=104 or n=62 for NPI) versus complete-case sensitivity analysis (excluding participants with incomplete diaries, n=62 for NPI-related outcomes).

**Outcome**	**Original Analysis**	**Complete-Case Sensitivity (n=62)**	**p-value (original vs sensitivity)**
NPI–RDI correlation (r_p)	0.38 (q=0.048)	0.41 (p=0.048)	–
ADH in severe vs non-severe OSA (pg/mL)	1.4 ± 0.8 vs 2.7 ± 1.1 (p=0.03)	1.3 ± 0.7 vs 2.6 ± 1.0 (p=0.029)	0.91
Severe RDI – IPSS total (p)	p=0.028	p=0.025	–
Severe RDI – nocturia episodes (p)	p=0.02	p=0.018	–
<60 years severe RDI – obstructive subscore (r_p)	0.96 (q=0.012)	0.95 (p=0.011)	–
≥60 years QoL–sleep efficiency (r_p)	0.48 (q=0.014)	0.49 (p=0.013)	–

Legend: All primary findings remained statistically significant and directionally unchanged after excluding participants with incomplete diaries, supporting robustness of the results despite 40% missing NPI data.
